# The Management of Tibial Bone Defects: A Multicenter Experience of Hexapod and Ilizarov Frames

**DOI:** 10.5435/JAAOSGlobal-D-23-00033

**Published:** 2023-08-03

**Authors:** Gerard A. Sheridan, Aaron Pang, Brian J. Page, Michael D. Greenstein, Gracielle S. Cardoso, Renato Amorim, S. Robert Rozbruch, Austin T. Fragomen

**Affiliations:** From the Limb Lengthening and Complex Reconstruction Service, Hospital for Special Surgery, New York, NY (Dr. Sheridan, Dr. Pang, Dr. Page, Greenstein, Dr. Rozbruch, and Dr. Fragomen), and the Serviço de Ortopedia e Traumatologia, Hospital Governador Celso Ramos, Florianópolis, Brazil (Dr. Cardoso and Dr. Amorim).

## Abstract

Bone defects may be managed with bone transport or acute shortening and lengthening using circular external fixation devices. We performed a multicenter retrospective cohort study to compare the outcomes between the Ilizarov frames and hexapod frames for the management of bone defects.

**Methods:**

Patients treated for bone defects using either Ilizarov or hexapod frames were included for analysis in two specialist institutions. Primary outcomes were time to consolidation, bone healing index (BHI), and external fixator index (EFI). Radiographic parameters included the medial proximal tibial angle, lateral distal tibial angle, posterior proximal tibial angle, and anterior distal tibial angle.

**Results:**

There were 137 hexapods and 90 Ilizarov frames in total. The mean time to follow-up was 3.7 years in the hexapod group and 4.0 years in the Ilizarov group. Hexapods had a significantly lower time to consolidation (253 days versus 449 days) (*P* < 0.0001) and BHI (59.1 days/cm versus 87.5 days/cm) (*P* < 0.0001). Hexapods had a significantly better EFI (72.3 days/cm versus 96.1 days/cm) (*P* = 0.0009).

**Conclusion:**

Hexapods may confer a significant advantage over Ilizarov frames in the management of bone defects. Time to consolidation, radiographic parameters, BHI, and EFI are all superior in hexapods.

The management of bone defects has long been a challenging area in the field of orthopaedic surgery. A myriad of options is available to the limb reconstruction surgeon managing these complex cases. Techniques available in this setting include bone transport, shortening and then lengthening, induced membrane technique, transport over a nail, free fibular grafting, and medial transport of the ipsilateral fibula.^1^

External fixation in this setting has been shown to be very effective in managing large bone defects up to the size of 18 cm and soft-tissue defects up to 30 × 35 cm in size.^2^ When applying the Ilizarov method to bone transport, union rates have been reported to be more than 90% with good functional and radiographic outcomes in most patients.^3^ In addition to managing bone defects, the Ilizarov method has been shown to be effective in the setting of soft-tissue injury that is not amenable to flap coverage. Intentional temporary limb deformation has been shown aid in the closure of soft-tissue defects that would not be possible without more complex soft-tissue reconstructive options.^4,5^

Over time, the evolution of external fixation has led to the genesis of the hexapod which has also been shown to be very effective in the setting of bone defect management.^6,7^ Functional outcomes after stacked hexapod application for bone transport can give good functional outcomes up to 8 years after initial surgery.^8^ When compared with Ilizarov frames, hexapods have been shown to be more powerful in the correction of translational and angular deformity, but many surgeons believe that hexapods are not stable enough to produce optimal regenerate when comparted with classic Ilizarov rigid ring connections.^9^ For this reason, comparative studies are important to tease out the optimal external fixation methods in this context.

To date, there have been no direct comparative studies assessing the differences between Ilizarov frames and hexapod frames in the management of tibial defects. In this study, we aim to assess the clinical and radiographic outcomes in the management of tibial defects. We performed a multicenter retrospective cohort study to compare the outcomes between the Ilizarov frames and hexapod frames for the management of tibial bone defects.

## Methods

This was a retrospective multicenter cohort study analyzing patients from two international institutions specializing in complex limb reconstruction. One institution exclusively used classic Ilizarov fixation methods, whereas the other used hexapod frames for management of these cases. The Ilizarov circular external fixators were stainless steel constructs and were connected with threaded rods using hinges when needed. In the Ilizarov cohort, both tensioned wires and half pins were used as bone fixation elements. The hexapod external fixators included the Taylor Spatial Frame (Smith & Nephew) and the MaxFrame (DePuy Synthes). Both institutions are centers of excellence for limb salvage and have outstanding reputations in using their respective techniques. Institutional Review Board approval was received before study commencement.

### Inclusion Criteria

All cases of tibial bone defect managed with either Ilizarov or hexapod external fixation were included for analysis. All cases underwent either bone transport or acute shortening and lengthening as the modality of surgical management. No time frame was specified for inclusion of cases to maximize the sample size for analysis.

### Data Collection and Minimum Data Set

The abovementioned data were collected for each patient. Clinical and functional outcome data were retrieved from retrospective chart review, and radiographic outcomes were recorded using local radiographic picture archiving and communication systems. A minimum data set was required for inclusion which included the following independent variables: age, sex, smoking status, Gustillo-Andersen classification of defect, bone defect size, fracture-related infection status, use of hexapod versus Ilizarov frame, and bone transport versus shortening and lengthening. Dependent outcome variables included the following: time to consolidation, external fixator index (EFI), bone healing index (BHI), radiographic regenerate quality (Modified Li score), radiographic alignment parameters (medial proximal tibial angle [MPTA], lateral distal tibial angle [LDTA], proximal posterior tibial angle [PPTA], and anterior distal tibial angle [ADTA]), and Association for the Study and Application of Methods of Ilizarov (ASAMI) bone criteria. Functional outcome measures included the ASAMI functional outcome score.

### Outcomes

Primary outcomes of interest were time to consolidation, BHI (which is a measure of the number of days taken to reach bone healing/consolidation per cm of defect size, unit—days/cm), and EFI (which is a measure of the number of days spent in the external fixator per cm of defect size, unit—days/cm). Radiographic outcomes included radiographic regenerate quality (Modified Li score),^10,11^ radiographic alignment parameters (MPTA, LDTA, PPTA, and ADTA), and ASAMI bone criteria. Functional outcomes included the ASAMI functional outcome score. Amputation was considered a failure of limb preservation and was documented for each case if performed.

## Statistics

Descriptive statistics were used to describe demographic data with interval variables being expressed as mean values with standard deviations and 95% confidence intervals (CIs) defined.

Univariate analysis for all outcomes of interest, where the independent variable was frame type (Ilizarov versus hexapod), was performed and all statistically significant associations were identified. The relationship between two categorical variables was analyzed using the chi-squared (χ^2^) test provided there were more than five subjects in each group. Otherwise, the Fisher exact test was used for these analyses. To determine the effect of categorical independent variables on interval dependent variables, the two-sample Student *t*-test with equal variances was used. The relationship between two interval variables was assessed using regression analyses, and regression plots were generated in these cases.

Based on the univariate analyses, multivariate regression analysis was performed to control for all confounding variables. A *P* value of 0.05 was taken to be statistically significant. Statistical software used for analysis was Stata/IC 13.1 for Mac (64-bit Intel).

## Results

In total, there were 137 hexapod frames and 90 Ilizarov frames included in this study for analysis. Table 1 summarizes the demographic characteristics of both the Ilizarov and hexapod group. Of note, the hexapod group was significantly older with a higher proportion of female patients compared with the Ilizarov group. In the hexapod group, most cases were managed with shortening and lengthening (62%), whereas in the Ilizarov group, most (57%) were managed using bone transport techniques. Mean follow-up times were comparable in both groups. The mean bone defect size was significantly larger in the Ilizarov cohort (6.73 cm; σ = 4.22; 95% CI, 5.84 to 7.61) compared with the hexapod cohort (5.02 cm; σ = 3.33; 95% CI, 3.89 to 6.15).

**Table 1:** Group Characteristic Comparison

Variable	Hexapod	Ilizarov	*P* Value
Sample	N = 137	N = 90	
Age	44.9 (σ = 13.5; 95% CI, 41.9-48.0)	38.6 (σ = 13.2; 95% CI, 35.8-41.4)	**0.0026**
Sex	71% male	83% male	**0.049**
Bone transport versus shortening + lengthening	38% BT, 62% S + L	57% BT, 43% S + L	**0.012**
Defect size (cm)	5.02 cm (σ = 3.33; 95% CI, 3.89-6.15)	6.73 cm (σ = 4.22; 95% CI, 5.84-7.61)	**0.031**
Mean time to follow-up (days)	1,350 (σ = 1,107; 95% CI, 1,088-1,612)	1,452 (σ = 818; 95% CI, 1,281-1,623)	*0.503*

BT = bone transport, CI = confidence interval, S + L = shortening and lengthening

### Clinical

**Figure 1:**
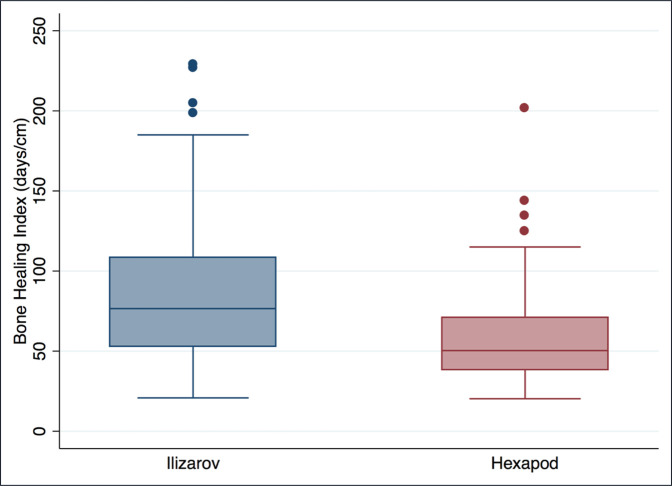
Box plot comparing bone healing index in the Ilizarov and hexapod group.

The time to consolidation was 253 days (σ = 124; 95% CI, 225 to 281) in the hexapod group and 449 (σ = 219; 95% CI, 403 to 495) in the Ilizarov group (*P* < 0.0001). The BHI was 59.1 days/cm (σ = 32.6; 95% CI, 51.6 to 66.6) in the hexapod group compared with 87.5 days/cm (σ = 46.6; 95% CI, 78 to 97) in the Ilizarov group (*P* < 0.0001) (Figure 1).

**Figure 2:**
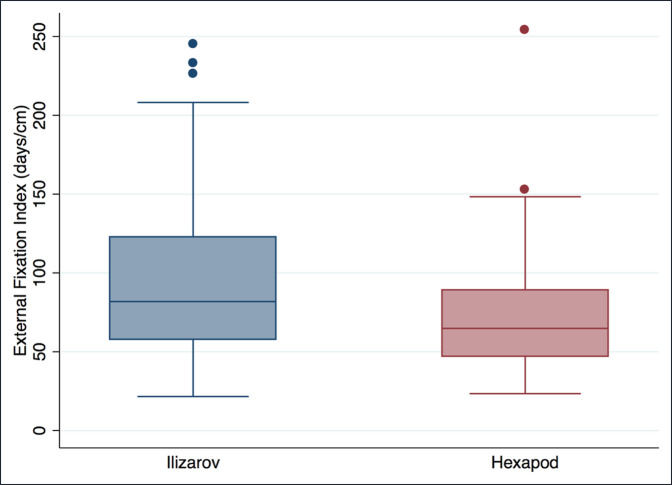
Box plot comparing external fixator index in the Ilizarov and hexapod group.

The EFI was 72.3 days/cm (σ = 36.4; 95% CI, 64.0 to 80.6) in the hexapod group and 96.1 days/cm (σ = 51.7; 95% CI, 85.3 to 107) in the Ilizarov group (*P* = 0.0009) (Figure 2).

For both groups, limb salvage was achieved in all cases. There were no amputations performed in either group at the time of follow-up.

### Radiographic

The regenerate quality as determined on postoperative radiographs was significantly better in the hexapod group (*P* = 0.003). The hexapod group had the following Modified Li scores: A: 66%, B: 30%, and C: 3% compared with the Ilizarov group with the following scores: A: 56%, B: 26%, and C: 19%.

Normal alignment parameters were achieved significantly more often in the hexapod group. All measures were better in the hexapod group with the exception of the LDTA which was not significantly different between both groups. The MPTA was normal in 83% of hexapods versus 61% in the Ilizarov group (*P* = 0.002). The LDTA was normal in 68% of hexapods compared with 57% in the Ilizarov group (*P* = 0.183). The PPTA was normal in 78% of hexapods compared with 57% in the Ilizarov group (*P* = 0.005). The ADTA was normal in 48% of hexapods compared with 31% in the Ilizarov group (*P* = 0.038).

The ASAMI bone score for the hexapod was excellent in 88% and good in 12%. In the Ilizarov group, 64% were reported as excellent, 28% as good, and 8% as poor (*P* = 0.0004).

### Functional

There was no significant functional difference between the hexapod and Ilizarov group using the ASAMI functional score (*P* = 0.54).

### Multivariate Analysis

After multivariate analysis, the only variable associated with an increased BHI after controlling for all confounders was frame type (*P* = 0.0018).

After multivariate analysis, the only variables associated with an increased EFI after controlling for all confounders were frame type (*P* = 0.044) and bone transport vs. shortening and lengthening (*P* = 0.0265).

## Discussion

This study is a retrospective comparison of the treatment of tibial bone defects with Ilizarov frames versus hexapod frames for either bone transport or acute shortening and lengthening. This study found that the hexapod group had statistically lower time to consolidation, lower BHI, and lower EFI when compared with the Ilizarov group. In addition, most radiographic parameters were significantly better in the hexapod group compared with the Ilizarov group (MPTA, PPTA, ADTA, regenerate quality using the Modified Li score, and ASAMI bone score). On multivariate analysis, the only significant predictor of improved BHI was the use of hexapod, and the only significant predictor of improved EFI was the use of a hexapod and the use of bone transport over shortening and lengthening. This information is of interest to surgeons because of the historical belief that regenerate quality was inferior using a hexapod frame compared with an Ilizarov frame. The current results have shown the hexapod to be more accurate (for final mechanical alignment achieved) and more efficient (for EFI and BHI) at managing these challenging bone defects. In addition, critical-sized bone defects of the tibia are complex and demanding problems that many surgeons encounter. Identifying the optimal external fixation technology to manage these conditions is of interest to all surgeons managing these complex cases. It should be mentioned that both methods of external fixation achieved limb salvage in all cases. Amputation was not performed in any case in either group. This indicates that both classic Ilizarov external fixation and hexapod management options are acceptable and viable options in the pursuit of limb salvage in this clinical context.

The Ilizarov method for treatment has been extensively reviewed in the literature and has been found to be a reliable option for managing infected and noninfected tibial bone defects.^3^ Aktuglu et al^3^ performed a narrative review of the available literature on this topic and found that patients with infected and noninfected critical sized tibial defects treated with the Ilizarov method had a low rate of poor bone formation and good functional results. Yin et al^12^ retrospectively reviewed 66 patients with tibial nonunions treated by bone transport with an Ilizarov frame and found that the external fixation index was 1.38 months/cm compared with 96.1 day/cm (roughly 3.2 months/cm) in this study. They found that radical débridement was the key step in achieving satisfactory bone formation and functional results. Miraj et al^13^ reviewed 14 patients with infected tibial nonunions with long devascularized and infected bone segments who had undergone several débridements, bone grafting, or spacer and soft-tissue closure. The patients then underwent radical resection and bone transport. They found that all patients had satisfactory results with a mean time to docking of 3.78 ± 0.54 months and a mean time to union of 7 (5.5 to 9) months. They had no recurrence of infection. This work would also support the continued use of classic Ilizarov external fixation for the management of major tibial bone defects. As demonstrated, no patients underwent amputation in the Ilizarov group, illustrating the effectiveness of this technique in achieving its ultimate goal of limb preservation.

Taylor spatial frames for the treatment of tibial bone defects have also been analyzed in the literature. Rozbruch et al^6^ reviewed 38 tibial nonunions (50% were infected) treated with a TSF. There were 23 patients with bone defects (average, 5.9 cm) and 22 patients with leg-length discrepancy (average, 3.1 cm). Treatment resulted in a final union rate of 95% with 11 patients requiring an additional procedure before union. This study shows an excellent time to union in the hexapod cohort with a BHI of 59.1 days/cm (σ = 32.6; 95% CI, 51.6 to 66.6).

Previous studies have aimed to compare Ilizarov and hexapod frames for various diagnoses, but this study is the largest cohort to date. Abuomira et al^14^ looked at 55 tibial nonunions comparing hexapods versus Ilizarov frames and found that consolidation at the docking site and the regenerate bone occurred in 49 of 55 (89%) cases and that there were no differences between the two groups. Direct comparisons of Ilizarov frames versus hexapod frames for treating fractures have also been studied. Tafazal et al^15^ looked at 10 patients treated with Ilizarov circular fixators, and 15 patients treated with Taylor spatial frames. They found both to be equally effective, but two cases of delayed union were reported in the TSF group. This may be in part because hexapod frame constructs are less rigid under axial loading but more rigid under bending and torsional loads compared with Ilizarov frames.^16^ Corona et al^17^ reviewed 31 patients analyzing fracture healing and infection eradication in acute fracture related infection comparing Ilizarov frames and hexapod frames. All patient's fractures healed, and all infections were eradicated. They found that the Ilizarov group had shorter time to fracture union and shorter duration in an external fixator, but the hexapod group had less residual coronal translation deformity and better callus quality. In this study, hexapods outperformed Ilizarov frames regarding time to consolidation, BHI, and EFI. Final limb alignment parameters were also markedly better in the Hexapod cohort.

There are many limitations to this study. Foremost, this is a retrospective study with inherent limitations in the variability of the data collected and the individuals' documenting encounters in the patients' chart. Hexapod technology was readily available at the limb reconstruction center in the United States, whereas the trauma center in Brazil did not have this hexapod technology available and so had to develop the advanced skill set needed to perform these surgeries with simple connecting elements as seen in the classic Ilizarov systems. Inherent in the international bias is the difference in patient demographic where compliance was high in the United States and more challenging in South America. We did not have the data available, but the extent of patient poverty (with concomitant social challenge) was far greater in the Brazilian cohort which can also shape treatment decisions. The Brazilians have become accustomed to leaving the frames on “longer than necessary” to prevent refracture in an unpredictable patient population. This socially driven clinical decision to leave frames on for “longer than necessary” clearly has a notable effect on the difference in EFI and BHI described in the above results section. In addition, it should be considered that the hexapod group was significantly older and comprised more female patients than the Ilizarov group. However, given the increased age of the hexapod group, one could expect poorer results, but this was not the case, further supporting the conclusion that hexapods may be superior to Ilizarov frames in this setting. There was also a significant difference in surgical indication between the two groups. There were more shortening plus lengthening in the hexapod group compared with bone transport in Ilizarov group. This may affect results, but there were still sizeable numbers of patients from both cohorts in the shortening and bone transport groups. The hexapod group and the Ilizarov group were operated on by different surgeons at different facilities. Surgeon preference imparts some degree of bias in all studies.

## Conclusion

This study found that hexapods may confer a significant advantage over Ilizarov frames in the management of bone defects treated with either bone transport or acute shortening and lengthening. We found that time to consolidation, BHI, and EFI were all superior in the hexapod group compared with the Ilizarov group. Final limb alignment parameters were also significantly better in the hexapod group. The only significant predictor of improved BHI was the use of hexapod. There were two significant predictors for lower EFI including the use of a hexapod and the use of bone transport over shortening and lengthening. This information is in direct contrast to previous studies and is of great interest to surgeons managing these complex reconstructive procedures. Although the results attained with the classic Ilizarov ring connections in expert hands were very good, this study supports the conclusion that the addition of hexapod technology will provide even better results in the treatment of bone defects.
